# Emergence of fluoroquinolone resistance and possible mechanisms in clinical isolates of *Stenotrophomonas maltophilia* from Iran

**DOI:** 10.1038/s41598-021-88977-z

**Published:** 2021-05-05

**Authors:** Akram Azimi, Farhad Rezaei, Mehdi Yaseri, Sirus Jafari, Mohammad Rahbar, Masoumeh Douraghi

**Affiliations:** 1 Division of Microbiology, Department of Pathobiology, School of Public Health, Tehran University of Medical Sciences, Poursina street, Enghelab-e-Eslami avenue, PO Box: 14155-6446, Tehran, Iran; 2 Virology Department, School of Public Health, Tehran University of Medical Sciences, Tehran, Iran; 3 Department of Epidemiology and Biostatistics, School of Public Health, Tehran University of Medical Sciences, Tehran, Iran; 4 Department of Infectious Diseases, Imam-Khomeini Hospital Complex, Tehran University of Medical Sciences, Tehran, Iran; 5 Reference Health Laboratories, Department of Microbiology, Ministry of Health, Tehran, Iran; 6 Food Microbiology Research Center, Tehran University of Medical Sciences, Tehran, Iran

**Keywords:** Antimicrobials, Microbial genetics

## Abstract

*Stenotrophomonas maltophilia* exhibits wide spectrum of fluoroquinolone resistance using different mechanisms as multidrug efflux pumps and Sm*qnr* alleles. Here, the role of *smeDEF*, *smeVWX* efflux genes and contribution of Sm*qnr* alleles in the development of fluoroquinolone resistance was assessed. Ciprofloxacin, levofloxacin and moxifloxacin resistance were found in 10.9%, 3.5%, and 1.6% of isolates, respectively. More than four-fold differences in ciprofloxacin MICs were detected in the presence of reserpine and *smeD, F, V* expression was significantly associated with ciprofloxacin resistance (p = 0.017 for *smeD*, 0.003 for *smeF*, and 0.001 for *smeV*). Sm*qnr* gene was found in 52% of the ciprofloxacin-resistant isolates and Sm*qnr8* was the most common allele detected. Fluoroquinolone resistance in *S. maltophilia* clinical isolates was significantly associated with active efflux pumps. There was no correlation between the Sm*qnr* alleles and ciprofloxacin resistance; however, contribution of the Sm*qnr* genes in low-level levofloxacin resistance was revealed.

## Introduction

Although *Stenotrophomonas maltophilia* have not been considered as a highly virulent pathogen^1^, more recently is known as one of the leading antibiotic-resistant pathogens in immunocompetent individuals^2^. Resistance of *S. maltophilia* strains to co-trimoxazole and ticarcillin-clavulanate which were recommended for empirical therapy was 4.7% and 16.1%, respectively around the world before 2003, but greater levels of resistance has now been reported and the trend of increasing antibiotic resistance is worrying^3^. In addition, *S. maltophilia* is intrinsically resistant to many commonly used antibiotics such as carbapenems and aminoglycosides and acquiring resistance to multiple antibiotics through plasmids, transposons, integrons result in the development of multi-drug resistant (MDR) strains, which makes it difficult to treat infections caused by this bacterium^4,5^.

Fluoroquinolones are antibiotics with a broad spectrum of antibacterial activity, have been used as an alternative therapeutic option against MDR *S. maltophilia* infections despite of their serious side effects and rapid resistance emergence on therapy^3,6^. Until recently, fluoroquinolones showed promising activity against *S. maltophilia*, but resistance to fluoroquinolones has currently been reported^7^. Resistance to fluoroquinolones is mainly attributed to mutations in chromosomal genes encoding, DNA gyrase and topoisomerase IV, and decreased intracellular concentration of quinolones as a result of porin alteration or overexpression of multidrug resistance (MDR) efflux pumps^8,9^. In addition, plasmid-mediated quinolone resistance (PMQR) has been found in Gram-negative bacteria and the genes responsible for such resistance are called *qnr* genes^10,11^. Multiple chromosomally encoded resistance determinants, including efflux pumps, antibiotic-inactivating enzymes and the quinolone resistance protein SmQnr have been considered as the mechanisms of antibiotic resistance in S. *maltophilia*^12,13^. However, *S. maltophilia* is the only known bacterium in which mutations in topoisomerases encoding genes is not associated with quinolone resistance^14^. Furthermore, *S. maltophilia* harbors a novel quinolone resistance gene, namely Sm*qnr* which is encoded by the chromosome, rather than plasmid-mediated *qnr* genes^15^. Therefore, development of resistance to quinolones and the relevant resistance mechanisms are not fully described in *S. maltophilia*^16^.

Despite the increasing prevalence of antibiotic resistance and a considerable resistance against fluoroquinolones (0–20%) in clinical isolates of *S. maltophilia* in Iran, mechanisms of fluoroquinolone resistance in Iranian isolates of *S. maltophilia* were not completely studied^17,18^. Here, the genetic background of resistance to fluoroquinolones including the role of active efflux pumps and their gene expression, the effect of reserpine as an efflux pump inhibitor on minimum inhibitory concentrations (MICs), and association of Sm*qnr* alleles with fluoroquinolone resistance in clinical isolates of *S. maltophilia* in Iran was sought.

## Results

### *S. maltophilia* strains

Among the 385 isolates collected, 375 were confirmed as *S. maltophilia* using phenotypic and genotypic methods and used for further experiments. These isolates were obtained from blood (n = 308), bronchoalveolar lavage (BAL) (n = 9), sputum (n = 5), wounds (n = 2), ascitic fluid (n = 2), respiratory secretions (n = 2), and other clinical sources (n = 47).

### Fluoroquinolone susceptibility of *S. maltophilia* strains

According to the disc diffusion method, ciprofloxacin resistance was found in 41 (10.9%) strains and among the remaining strains, 113 (30.1%) showed intermediate susceptibility to ciprofloxacin and 221 (58.9%) were ciprofloxacin-susceptible. Thirteen (3.5%) and 6 (1.6%) strains showed resistance or intermediate susceptibility to levofloxacin. Majority of the strains was susceptible to moxifloxacin (369, 98.4%), one strain was intermediate susceptible and only five strains (1.3%) were resistant to moxifloxacin. Table 1 shows the susceptibility profile of the *S. maltophilia* strains to ciprofloxacin and levofloxacin. Based on the MICs, 48 ciprofloxacin- and 4 levofloxacin- resistant strains were identified with the MICs equivalent or greater than 2 and 8 µg/mL, respectively. Furthermore, MIC90 of ciprofloxacin was ≤ 32 µg/mL while that was ≤ 2 µg/mL for levofloxacin.

**Table 1:** Minimum inhibitory concentration ranges and susceptibility pattern of *Stenotrophomonas maltophilia* strains against ciprofloxacin and levofloxacin.

Antibiotic	Susceptibility pattern (MIC range)	No. of strains (%)	MIC_50_	MIC_90_
			(µg/mL)	
Ciprofloxacin	Susceptible (≤ 0.5)	0 (0%)	≤ 4	≤ 32
	Intermediate (1)	2 (4%)		
	Resistant (≥ 2)	48 (96%)		
Levofloxacin	Susceptible (≤ 2)	46 (92%)	≤ 0.5	≤ 2
	Intermediate (< 2, > 8)	0 (0%)		
	Resistant (≥ 8)	4 (8%)		

Since the critical concentrations of ciprofloxacin for *Stenotrophomonas maltophilia* are not defined by the CLSI, the critical concentrations of *Pseudomonas aeruginosa* have been used to interpret the results of ciprofloxacin susceptibility^35^.

*MIC* minimum inhibitory concentration.

### Effect of reserpine on ciprofloxacin MICs

**Figure 1:**
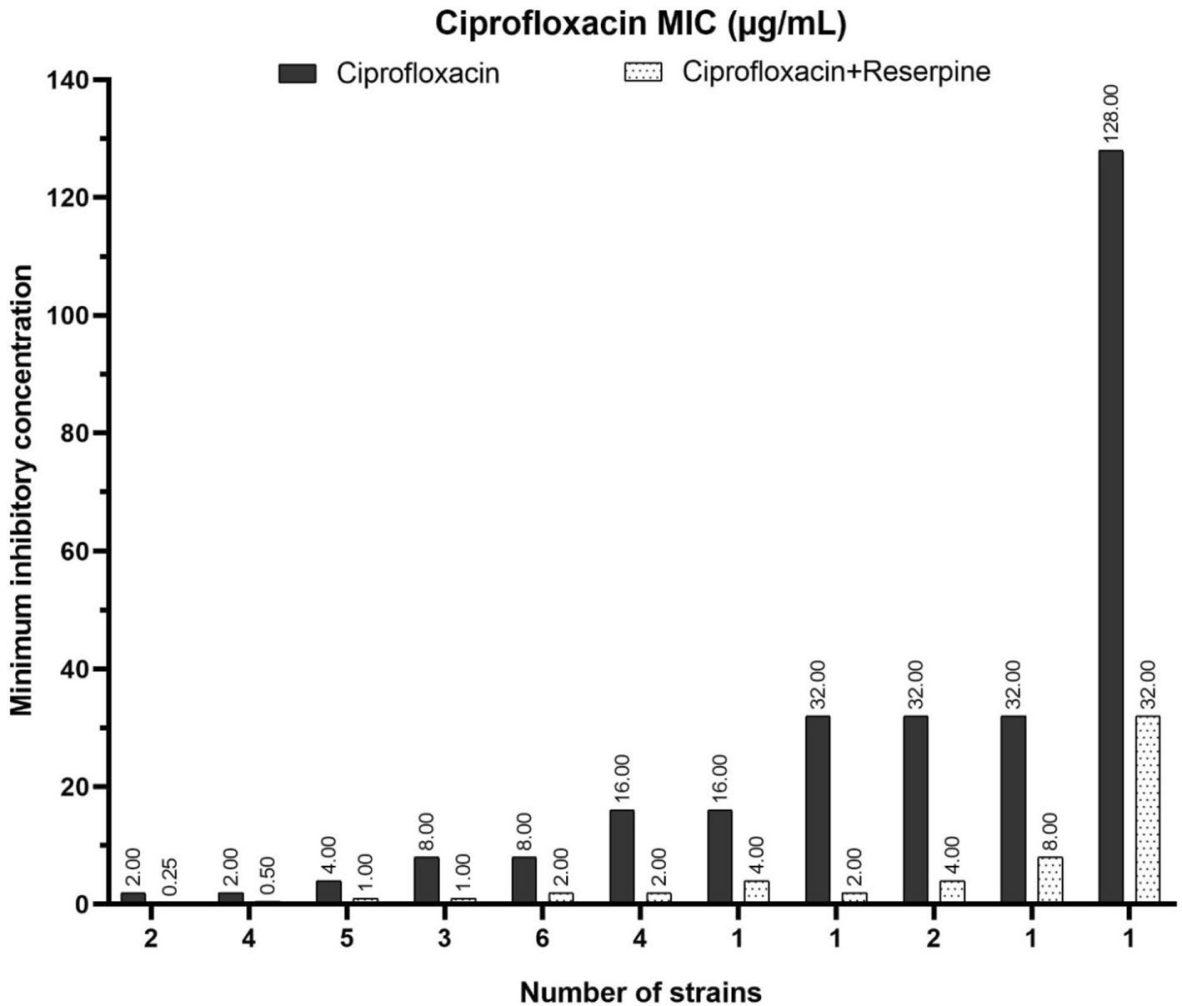
Minimum inhibitory concentrations of ciprofloxacin among the 30 strains of *Stenotrophomonas maltophilia* before and after the reserpine treatment.

MICs of ciprofloxacin were reduced in 30 out of 48 ciprofloxacin-resistant strains following reserpine treatment from 4 to 16 folds indicating active efflux pump in these strains. Among them, 6 resistant strains became susceptible, 8 resistant strains identified as intermediate susceptible to ciprofloxacin and 16 resistant strains showed decreased MICs of ciprofloxacin. Strains with active efflux pumps showed significantly greater MICs of ciprofloxacin (*p* = 0.001) but not levofloxacin (*p* = 0.081). The MICs of ciprofloxacin with and without reserpine among the 30 *S. maltophilia* strains with reduced MICs are shown in Fig. 1.

### The presence of *smeDEF* and *smeVWX* genes

Among the 48 strains ciprofloxacin-resistant strains, *smeE* and *smeF* genes were not detected in 3 and 8 strains, respectively using PCR. The remaining strains yielded amplicons for *smeD*, *smeE*, *smeF*, *smeV*, *smeW* and *smeX* genes (Supplementary Table S1). There were not significant differences in the MICs of ciprofloxacin/levofloxacin among the *smeDEF*-positive and s*meDEF*-negative *S. maltophilia* strains (p > 0.05).

### Expression of *smeD*, *smeF* and *smeV* genes

**Figure 2:**
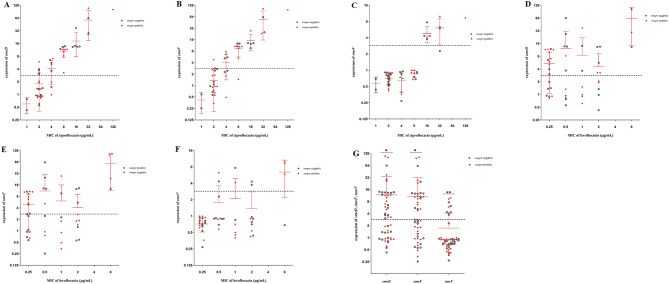
Expression level of *smeD*, *smeF*, and *smeV* genes in *Stenotrophomonas maltophilia* strains. (**A**–**C**) *smeD*, *smeF*, and *smeV* expression level and distribution of ciprofloxacin MICs, (**D**–**F**) *smeD*, *smeF*, and *smeV* expression level and distribution of levofloxacin MICs, (**G**) the mean expression level of *smeD*, *smeF*, and *smeV* genes among the *S. maltophilia* isolates. Red lines showing the mean ± SD for each group and the dash line indicates the level of gene expression above which overexpression is considered, *expression level of *smeD* (p = 0.01) and *smeF* (p = 0.003) genes was significantly associated with the reduced MICs of ciprofloxacin, **the corelation of *smeV* expression level with the MICs of both ciprofloxacin (p = 0.000) and levofloxacin (p = 0.03) was significant.

Twenty-nine out of 48 ciprofloxacin-resistant strains were detected with ≥ threefold expression of *smeD* gene. Compared to the *S. maltophilia* ATCC13637, overexpression of *smeF* gene was found in 24 strains and expression level of *smeV* gene was ≥ 3 folds in 9 strains. Overexpression of the three efflux pump genes tested were noted in 3 out of 4 levofloxacin-resistant strains and one levofloxacin-resistant strain showed overexpression for *smeD* and *smeF* genes but not *smeV*. The expression of *smeD*, *F*, *V* genes was significantly correlated with higher MICs of ciprofloxacin and this correlation was also found between *smeV* gene and levofloxacin, compared to ATCC13637 standard strain. The expressions level of *smeD*, *smeF*, and *smeV* genes are demonstrated in Fig. 2.

### Sm*qnr* alleles

**Figure 3:**
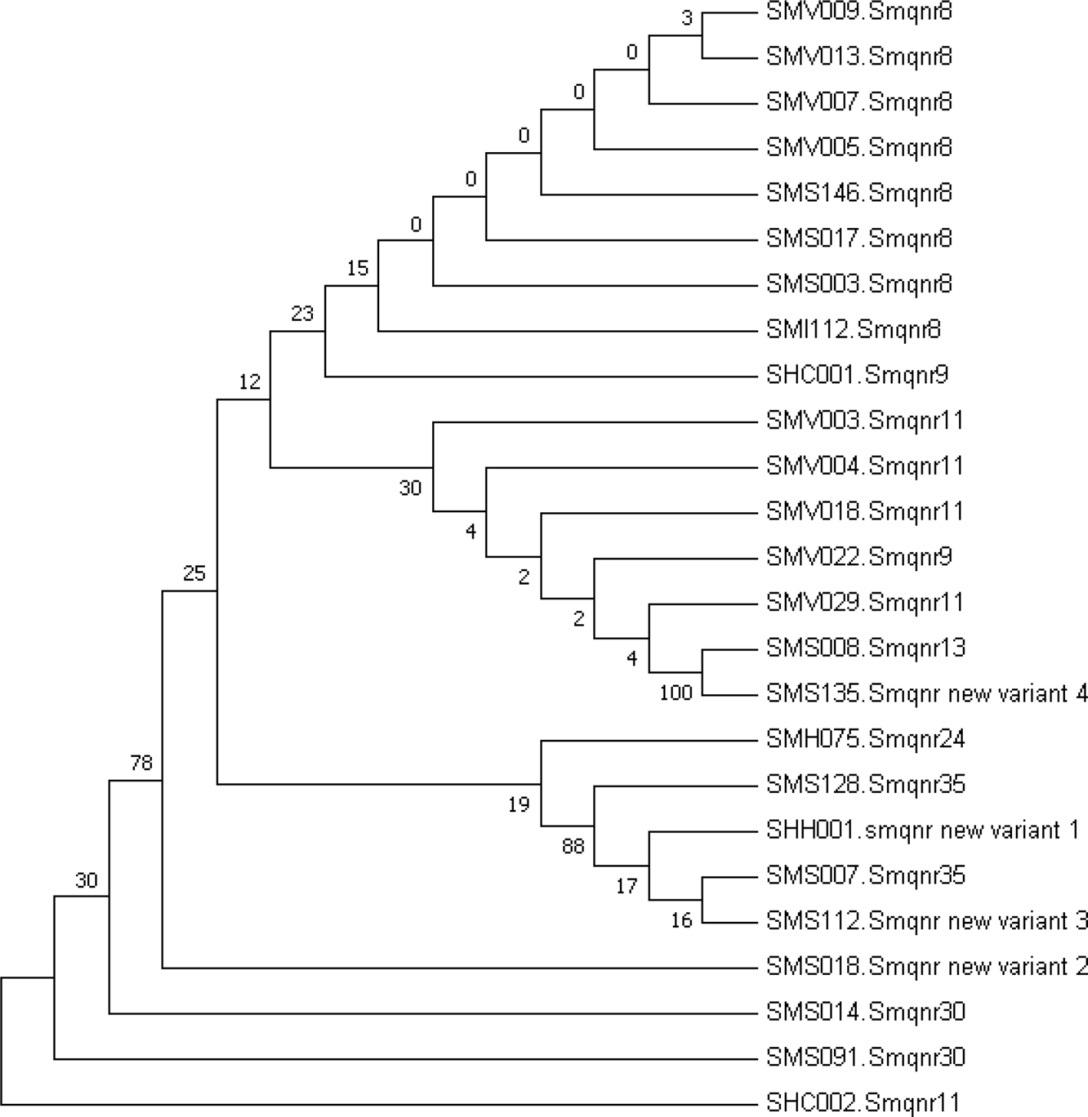
Phylogenetic tree of 4 new and already known Sm*qnr* alleles in *Stenotrophomonas maltophilia* strains tested in this study. The evolutionary history was inferred using the Neighbor-Joining method. The optimal tree with the sum of branch length = 3.10403504 is shown. The percentage of replicate trees in which the associated taxa clustered together in the bootstrap test (1000 replicates) are shown next to the branches. The evolutionary distances were computed using the Poisson correction method and are in the units of the number of amino acid substitutions per site.

The Sm*qnr* gene was identified in 25 strains of 48 ciprofloxacin- resistant with the following allele distribution: Sm*qnr8* (n = 8), Sm*qnr9* (n = 2), Sm*qnr11* (n = 5), Sm*qnr13* (n = 1), Sm*qnr24* (n = 1), Sm*qnr30* (n = 2), Sm*qnr35* (n = 2), and 4 distinct new alleles, hereafter named new variant -1, -2, -3, and -4 (Supplementary Fig. S1). Differences in amino acid sequences among the 4 new variants are as follows: new variant 1 (R95H, 99.5% identity to Sm*qnr35*), new variant 2 (T64A, 99.5% identity to Sm*qnr40*), new variant 3 (Q23E and Q28H substitutions with 99.1% identity to Sm*qnr35*), and new variant 4 (L161R, with identity of 99.5% to Sm*qnr13*). Three levofloxacin-resistant strains with MIC equal to 8 µg/mL carried Sm*qnr9* (n = 2) and the new variant-4 (n = 1). A levofloxacin-resistant strain was Sm*qnr* negative. The sequence alignment of the all subtypes and amino acid substitutions of Sm*qnr* are shown in Supplementary Fig. S1. Phylogenetic tree of the 4 new and 21 known Sm*qnr* alleles and their relative distances are shown in Fig. 3. Three major clusters were found. Two new variants (1 and 3) were classified in a cluster alongside with known Sm*qnr -24, -35* and the new alleles 4 and known Sm*qnr -9, -11, and -13* were concentrated in another cluster.

### Co-effect of efflux pumps and Sm*qnr* alleles on ciprofloxacin MICs

Based on the expression of efflux pump genes and/or presence of Sm*qnr* alleles, the isolates were classified into the following sets: (a) the isolates which harbored Sm*qnr* alleles and had *smeDEF* overexpression (14 isolates); 57.1% (4 isolates) of these isolates had MIC ≥ 4 µg/mL but the differences of ciprofloxacin MICs among the isolates in this group was not statistically significant compared to the isolates without efflux pump genes overexpression and Sm*qnr* alleles (p = 0.08), (b) the isolates having *smeVWX* overexpression and Sm*qnr* alleles (four isolates); all these were among the resistance isolates and there were no significant differences in the ciprofloxacin MICs of isolates with *smeVWX* overexpression and Sm*qnr* alleles in comparison with the isolates which did not have efflux pump genes overexpression and Sm*qnr* alleles (p < 0.05), (c) the isolates with overexpression of *smeDEF* and *smeVWX* and Sm*qnr* alleles(four isolates); all the isolates in this category showed MIC ≥ 4 µg/mL. The comparison of ciprofloxacin MICs between this recent group and the isolates with no overexpression of efflux pump genes and Sm*qnr* alleles was significant (p = 0.002).

## Discussion

Intrinsic resistance nature of *S. maltophilia* against multiple antimicrobial agents and limited therapeutic options made great concern to control the increasing *S. maltophilia* nosocomial infections. However, resistance rate of *S. maltophilia* strains varies depending on different geographical areas. In this study, a large series of isolates from Tehran and a neighboring province were studied and we found a susceptibility rate of fluoroquinolones (89% for ciprofloxacin and 96.5% for levofloxacin) similar to the previous study (84.1% for ciprofloxacin and 99.4% for levofloxacin) in Iran which studied 44 and 45 isolates^18,19^. The resistance to ciprofloxacin ranged from 13 to 96% globally^3^ and fluoroquinolone resistance in neighboring countries of Iran was as follows: an increasing rate of resistance from 7.8% (1998–2003)^20^ to 89% (2006–2013)^21^ in Turkey, 64–100% in Pakistan^22^ and 23% (2003–2009) in Saudi Arabia^23^.

Overexpression of *smeDEF* and *smeVWX* genes might contribute to increased MICs of multiple antibiotics and developing multi-drug resistant *S. maltophilia* strains^24^. In this study, the average of increased MICs of ciprofloxacin in *smeD, F, V* overexpressed strains was statistically significant compared to the isolates with no overexpression. The differences of MICs of levofloxacin were statistically significant only when the *smeV* gene is overexpressed. Furthermore, the differences of MICs of ciprofloxacin in the presence of reserpine confirmed that ciprofloxacin resistance was affected by the *smeD, F, V* overexpression. Therefore, the significant role of active efflux in fluoroquinolone resistance of *S. maltophilia* strains was demonstrated in the present study. These findings are supported by the results from previous studies^24–26^. In contrast, Wu *et al*. demonstrated that *smeDEF* did not considerably contribute to fluoroquinolone resistance and implication of efflux pumps in resistance to fluoroquinolone might have overestimated^27^.

There were geographical differences of 47–70% in Sm*qnr* frequency^28^ and little correlation between the Sm*qnr* alleles and resistance to fluoroquinolones in the *S. maltophilia* isolates was assumed^29^. Nonetheless, almost half (n = 25, 52.1%) of the 48 ciprofloxacin-resistant *S. maltophilia* strains (MIC ≥ 2 µg/mL) were found carrying Sm*qnr* genes in the present study. Two remaining strains with intermediate susceptibility to ciprofloxacin (MIC = 1 µg/mL) was Sm*qnr*-negative. Four strains with high level resistance to both ciprofloxacin (MIC = 8, 32 (2 isolates), and 128 µg/mL) and levofloxacin (MIC = 8 µg/mL) showed overexpression for *smeDF* genes harboring Sm*qnr9* (n = 2), Sm*qnr* new variant 4 (n = 1), and a Sm*qnr*-negative strain. The frequency of 65.9% Sm*qnr* alleles were also previously reported in*S. maltophilia* strains from Iran which studied only 44 strains^18^. The most common Sm*qnr* allele in the current study was Sm*qnr*8, followed by Sm*qnr*11, Sm*qnr*9, Sm*qnr*30, and Sm*qnr*35. Therefore, there was no significant association between ciprofloxacin resistance and Sm*qnr* alleles in the strains examined (p = 0.2). However, significant differences of levofloxacin MICs among the Sm*qnr*-positive and Sm*qnr*-negative strains were found (p = 0.008). Significant difference in resistance to levofloxacin of Sm*qnr*-positive isolates was previously demonstrated by Kanamori *et al.* using a MIC of ≥ 2 µg/mL for levofloxacin but not a MIC of ≥ 8 µg/mL and they highlighted the role of Sm*qnr* genes in low-level fluoroquinolone resistance^28^. The isolates with the Sm*qnr*8 and Sm*qnr*11 were found to be levofloxacin-intermediate susceptible; however, three of 8 isolates with Sm*qnr*8 and 3 of 5 isolates having Sm*qnr*11 alleles were found among the ciprofloxacin-resistant isolates (MIC ≥ 2 µg/mL). The two isolates with Sm*qnr9* allele and a Sm*qnr* new variant-4 positive strain were resistant to both ciprofloxacin (MIC ≥ 2 µg/mL) and levofloxacin (MIC ≥ 8 µg/mL). Three Sm*qnr* alleles including Sm*qnr*24, 30, and new variant-1 were found among the isolates with intermediate susceptibility levofloxacin. Totally, 22 (88%) out of 25 Sm*qnr* positive isolates were intermediate susceptible to levofloxacin and this might propose that Sm*qnr* alleles were mostly related to low-level fluoroquinolone resistance as Kanamori *et. al.* reported^28^. The role of Sm*qnr* genes remains obscure and high-level fluoroquinolone resistance in *S. maltophilia* isolates might be associated with mechanisms other than Sm*qnr* as described previously^29,30^.

Comparison of the MICs of ciprofloxacin in the presence of both efflux and Sm*qnr* alleles showed that the higher MICs were noted when overexpression of the two *smeDEF* and *smeVWX* efflux pump genes alongside with the Sm*qnr* alleles detected. Totally, the higher MIC levels more related with two parameters; the number of overexpressed genes and the level of expression (higher levels of expression in more genes result in higher MICs). Therefore, according to these findings and comparison with the results of the isolates only having Sm*qnr* alleles or active efflux pumps, overexpression of *smeDEF* and *smeVWX* genes were more important in resistance development and can lead to high level fluoroquinolone resistance. High-level fluoroquinolone resistance due to the overexpression of multi-drug efflux pump *semDEF* and low-level fluoroquinolone resistance by *qnrD* were already reported by Cavaco *et al*. and Valdezate *et al*.^31,32^.

In conclusion, this study revealed that active efflux pumps can significantly contribute in fluoroquinolone resistance in *S. maltophilia* isolates. No correlation between the Sm*qnr* alleles and ciprofloxacin resistance in the clinical isolates of *S. maltophilia* was found, but Sm*qnr* alleles were mostly associated with lower MICs of levofloxacin. Therefore, efflux pumps were largely linked to higher MICs of fluoroquinolone than the Sm*qnr* alleles. Further studies are required to assess the contribution of Sm*qnr* to the fluoroquinolone susceptibility of *S. maltophilia* isolates.

## Materials and methods

### Bacterial isolates and identification

A total of 385 clinical isolates of *S. maltophilia* were collected during the period between September 2010 and August 2017 from six hospitals (H1–H4, H6, H11) in Iran. Phenotypic identification of the isolates was done using different biochemical tests including, oxidase, catalase, DNase, nitrate reduction, citrate, esculin hydrolysis, gelatin liquefaction, lysine decarboxylase and sugar fermentation on triple sugar iron (TSI) agar^5^. Genomic DNA was prepared from single colony of each isolate using the standard phenol–chloroform method^33^ and species-specific PCR (SS-PCR) using the following primers; SM1 5′-CAGCCTGCGAAAAGTA-3′ and SM4 5′-TTAAGCTTGCCACGAACAG-3′ was applied to target the *23S rRNA* gene^34^. Gel electrophoresis was done to confirm the presence of the amplicons of 531 bp in *S. maltophilia* strains^34^. A representative amplicon of *23S rRNA* gene was subjected to sequencing and the sequence was deposited in GenBank under the accession no. JQ889327 (https://www.ncbi.nlm.nih.gov/nuccore/JQ889327).

### Antimicrobial susceptibility testing

Antibiotic susceptibility testing of the isolates against ciprofloxacin (5 µg), levofloxacin (5 µg) and moxifloxacin (5 µg) (Mast Group Ltd, UK) was determined using the disk diffusion method on the Mueller–Hinton agar (Merck, Germany) plates according to Clinical and Laboratory Standards Institute (CLSI) guidelines^35^. In addition, minimum inhibitory concentrations (MICs) of ciprofloxacin and levofloxacin were determined by broth microdilution method for the isolates which were considered as ciprofloxacin-intermediate/-resistant according to the disk diffusion method. In other words, 50 isolates were selected for this experiment, in which 41 isolates were ciprofloxacin resistant and the remaining nine isolates were among the ciprofloxacin-intermediate-susceptible isolates which were selected based on isolation date, sources, the presence of resistance genes and MICs). In brief, twofold serial dilutions of ciprofloxacin and levofloxacin were prepared in 96-well microplates containing Mueller–Hinton broth (Merck, Germany) to obtain the concentration ranging from 0.25 to 128 μg/mL. The 0.5 MacFarland bacterial suspensions were used and the final concentration was equal to 5 × 10^5^ CFU/mL. The plates were sealed and incubated for 20–24 h at 35 °C. The critical breakpoints of ciprofloxacin for *Pseudomonas aeruginosa* were used for interpretation of the results because of no breakpoints for *S. maltophilia* were recommended by the CLSI^35^ and the results of moxifloxacin were interpreted according to the British Society for Antimicrobial Chemotherapy (BSAC) guidelines^36^. The *S. maltophilia* ATCC 13637 and *Pseudomonas aeruginosa* ATCC 27853 were used as quality control strains.

### MIC determination in the presence of reserpine

The MICs of ciprofloxacin were determined in the presence of an efflux pumps inhibitor, ion motive ATPase; reserpine (Sigma Aldrich, St. Louis, MO, USA). The broth microdilution method was performed as described above with a final concentration of 25 μg/mL reserpine in the Cation-adjusted Mueller Hinton broth (Merck, Germany)^37^. A change of four-fold or higher, in the ciprofloxacin MICs with and without reserpine was considered as inhibition of active efflux of the drug^38^.

### Genomic detection of *smeDEF* and *smeVWX* genes

To confirm the presence of the genes encoding efflux pumps including, *smeDEF* and *smeVWX*, PCR was done using specific primers for *smeD*, *smeE*, *smeF*, *smeV*, *smeW*, and *smeX* genes (Table 2). A representative PCR amplicon of each gene was sequenced to ensure the specific amplification.

**Table 2:** Primers used in PCR detection of *smeDEF* and *smeVWX* genes in *Stenotrophomonas maltophilia* strains.

Gene	Oligonucleotide sequence (5′ to 3′)	Tm (°C)	Amplicon size (bp)	References
***smeD***				
F	CCAAGAGCCTTTCCGTCAT	57.5	150	^25^
R	TCTCGGACTTCAGCGTGAC	59.5		
***smeE***				
F	AGCTCGACGCCACGGTA	57.3	803	^25^
R	TGGCCTGGATCGAGAGCA	58.4		
***smeF***				
F	GCCACGCTGAAGACCTA	54.9	800	^25^
R	CACCTTGTACAGGGTGA	52.4		
***smeV***				
F	GTCGACTTCCTCGACAACC	59.5	212	^39^
R	TTGCCATCCTTGTCTACCAC	58.4		
***smeW***				
F	GCCCACACCATCTCGTTCCC	64.6	221	^40^
R	TAGCCGTTGCCGTTGCCC	60.8		
***smeX***				
F	TACGACCGCCGCAAGCAACC	64.6	219	^40^
R	CAGCTCGAAGTAGTTGCGTGCC	65.8		

*F* forward, *R* reverse.

### Quantitative reverse transcription PCR (RT-qPCR)

A single colony of each isolates were cultured in Luria–Bertani (LB) broth (Merck, Germany) and placed in a 37 °C shaking incubator at 180 rpm until the growth reached logarithmic phase (OD600 = 0.5). The log-phase bacterial cell were used to extract total RNA by the RNeasy mini kit (Qiagen, Valencia, CA, USA) according to the manufacturer’s instructions. Then, total RNA was treated with RNase free DNase I (Ambion, Austin, TX, U.S.A.) to further eliminate genomic DNA. After that, the quantity and quality of yielded RNA were evaluated using the Nanodrop (Thermo Scientific, Waltham, MA, USA) and RNA integrity verification was done on 1% agarose gel. Finally, the purified RNA was confirmed by PCR using *gyrA* primers (Table 3). The StepOnePlus™ real-time PCR System (Applied Biosystems, Foster City, CA, USA) was used to perform the relative RT-qPCR on the synthesized cDNA (PrimeScript RT reagent kit (Parstous, Iran) using specific primers for *smeD*, *smeF*, and *smeV* genes (Table 3). Each reaction mixture contains 8 μL of the Power SYBR Green PCR Master Mix (Bioneer, Korea), 1 μL of each primer (10 pM), and 2 μL of cDNA in a final volume of 20 μL by adding distilled water and the RT-qPCR was run under the following conditions: initial denaturation of 10 min at 95 °C, 40 cycles of 95 °C for 20 s and 61 °C for 40 s followed by melting curve analyses to ensure specific amplification. This experiment was run in triplicate (from the same sample) for all isolates tested.

**Table 3:** Primers used in relative Rt qPCR for amplification of *smeD*, *smeF*, *smeV*, and *gyrA* genes in *Stenotrophomonas maltophilia* strains.

Gene	Oligonucleotide sequence (5′ to 3′)	Tm (°C)	Amplicon size (bp)	References
***gyrA***				
F	CCAGGGTAACTTCGGTTCGA	60.5	60	^41^
R	GCCTCGGTGTATCGCATTG	59.5		
***smeD***				
F	CCAAGAGCCTTTCCGTCAT	57.5	150	^25^
R	TCTCGGACTTCAGCGTGAC	59.5		
***smeF***				
F	TCGTCCAGGCTGACATTCAA	58.4	101	^25^
R	AACGCGGATCGTGATATCG	57.5		
***smeV***				
F	GTCGACTTCCTCGACAACC	59.5	212	^39^
R	TTGCCATCCTTGTCTACCAC	58.4		

The RT-qPCR data analysis was carried out using the 2^−∆∆CT^ method to evaluate expression level of *smeD*, *smeF* and *smeV* genes by normalization to the *gyrA* housekeeping gene as well as compared to the *S. maltophilia* ATCC 13637 as a reference strain. Efflux pump expression greater than 3 folds was considered as overexpression^27^.

### PCR detection and sequence analysis of Sm*qnr* alleles

To amplify a 811 bp fragment of Sm*qnr* gene, the following primer set was used; forward primer; 5′-ACACAGAACGGCTGGACTGC-3′ and reverse primer; 5′-TTCAACGACGTGGAGCTGT-3′^29^. PCR was performed using the 10 µL of *Pfu* PCR PreMix, (Bioneer, Korea), 10 pM of each primer, 50 ng of template DNA and 6 µL of distilled water to reach final volume of 20 µL PCR reaction mix. PCR products were sequenced with the corresponding PCR primers and translated to amino acid sequences using the Expasy translate tool (http://web.expasy.org/translate/). The obtained sequences were compared to the previously deposited Sm*qnr* sequences in GenBank and alleles with one or more amino acid substitution were considered as new variants^6^. Multiple alignments of all available Sm*qnr* sequences in GenBank till October 14, 2020 were performed to analyze the phylogenetic relationships of Sm*qnr* alleles. The phylogenetic tree of Sm*qnr* genes was constructed using Molecular Evolution and Genetic Analysis (MEGA) version 7.0.14 (http://www.megasoftware.net/).

### Nucleotide accession numbers

The sequences of Sm*qnr* genes have been submitted to the GenBank and the assigned accession numbers are as follows: Sm*qnr*8 (MT920916, MT920917, MT997012, MT997013, MT997019, MT997020, MT997021, MT997023), Sm*qnr*9 (MT997015, MT861992), Sm*qnr*11 (MT920914, MT920915, MT921276, MT920913, MT997025), Sm*qnr*13 (MT920912), Sm*qnr*24 (MT890701), Sm*qnr*30 (MT997016, MT997017), Sm*qnr*35 (MT928300, MT997014) and new Sm*qnr* (MT939666, MT997026, MT997027, MT997028).

### Statistical analysis

The SPSS version 23.0 was used to analyze the obtained results. Descriptive statistics of the data was conducted by frequencies and crosstabs. The Pearson Chi-Square test was used to analyze the reduction of ciprofloxacin MICs under reserpine treatment. The effect of the presence or absence of efflux pump genes on antibiotic resistance was evaluated using Kruskal–Wallis test. The correlation of the antibiotic MICs with the efflux pump’s expression or Sm*qnr* alleles was evaluated using Pearson Chi-Square test. co-effect of efflux pumps and Sm*qnr* alleles on ciprofloxacin MICs was assessed by Chi-Square test. The MIC_50_ (MIC required to inhibit the growth of 50% of organisms) and MIC_90_ (MIC required to inhibit the growth of 90% of organisms) of ciprofloxacin and levofloxacin of the strains were calculated. A p value of < 0.05 was considered significant.

### Ethics approval

This study was approved by the Ethics Committee of Tehran University of Medical Sciences “IR. TUMS. MSP. SPH. REC.1396.4388”.

## Supplementary Information

Supplementary Table S1.

Supplementary Figure S1.
