# Supplementary material for: Emergence of fluoroquinolone resistance and possible mechanisms in clinical isolates of Stenotrophomonas maltophilia from Iran
Source: Sci Rep. 2021 May 5;11:9582. doi: 10.1038/s41598-021-88977-z (PMC8100118; doi:10.1038/s41598-021-88977-z)
Supplement: Supplementary file 1 — Supplementary Table S1. [file 41598_2021_88977_MOESM1_ESM.docx]

**Supplementary Table 1**. Drug susceptibility, genomic detection of efflux pumps and their expression, and effect of reserpine treatment on the minimum inhibitory concentrations of 50 *Stenotrophomonas maltophilia* isolates with susceptibility, intermediate or resistance to ciprofloxacin.

| No. | ID | Date | Source | Genomic detection | | | | | | Expression (mean ± SD) | | | Sm*qnr*  allele | MIC^a^ | | |
| --- | --- | --- | --- | --- | --- | --- | --- | --- | --- | --- | --- | --- | --- | --- | --- | --- |
|  |  |  |  | *smeD* | *smeE* | *smeF* | *smeV* | *smeW* | *smeX* | *smeD* | *smeF* | *smeV* |  | CIP^b^ | CIP+Res^c^ | LEV^d^ |
| Standard strain | SM13637 |  |  | + | + | + | + | + | + | 0.99 | 0.97 | 0.84 | - | 0.25 | 0.25 | 0.25 |
| 1 | SIH008 | Sep-2010 | Neurology | + | + | + | + | + | + | 3.99 ± 0.4 | 2.81 ± 0.2 | 0.91± 0.1 | - | 2 | 0.5 | 0.25 |
| 2 | SIH014 | Nov-2010 | Pediatrics | + | + | + | + | + | + | 0.98 ± 0.1 | 0.56 ± 0.1 | 0.70 ± 0.1 | - | 2 | 2 | 2 |
| 3 | SHC001 | Oct-2010 | ICU | + | + | + | + | + | + | 109.21 ± 0.4 | 103.47 ± 0.3 | 5.84 ± 0.6 | *smqnr9* | 32 | 4 | 8 |
| 4 | SHC002 | Nov-2010 | C3 | + | + | + | + | + | + | 2.07 ± 0.08 | 1.82 ± 0.2 | 0.89 ± 0.2 | *smqnr11* | 2 | 0.5 | 2 |
| 5 | SMH075 | Jan-2011 | Pediatric | + | + | + | + | + | + | 0.85 ± 0.01 | 0.04 ± 0.005 | 0.90 ± 0.1 | *Smqnr24* | 2 | 2 | 0.5 |
| 6 | SMH092 | Mar-2011 | Neurosurgery | + | + | + | + | + | + | 3.64 ± 0.5 | 2.26 ± 0.03 | 0.85 ± 02 | - | 4 | 1 | 1 |
| 7 | SHH001 | Jan-2012 | NR^g^ | + | - | + | + | + | + | 0.61 ± 0.08 | 0.34 ± 0.001 | 0.50 ± 0. 1 | New variant 1 | 2 | 2 | 1 |
| 8 | SMH103 | Nov-2012 | NR | + | + | + | + | + | + | 13.28 ± 0.5 | 12.42 ± 0.4 | 3.76 ± 0.1 | - | 16 | 4 | 2 |
| 9 | SMS002 | Aug-2016 | Emergency | + | + | + | + | + | + | 3.69 ± 0.02 | 2.46 ± 0.09 | 0.87 ± 0.1 | - | 2 | 0.5 | 0.5 |
| 10 | SMS003 | Sep-2016 | ICU | + | + | - | + | + | + | 8.17 ± 0.4 | 5.18 ± 0.05 | 0.90 ± 0.04 | *smqnr8* | 8 | 2 | 0.5 |
| 11 | SMS006 | Sep-2016 | Blood disorders | + | + | + | + | + | + | 0.56 ± 0.2 | 0.01 ± 0.006 | 0.57 ± 0.1 | - | 2 | 2 | 0.5 |
| 12 | SMS007 | Sep-2016 | Neurosurgery | + | + | + | + | + | + | 11.43 ± 0.6 | 10.39 ± 0.4 | 0.99 ± 0.04 | *smqnr35* | 8 | 1 | 0.25 |
| 13 | SMS008 | Sep-2016 | Respiratory | + | + | + | + | + | + | 37.12 ± 0.5 | 27.38 ± 0.7 | 4.11 ± 0.8 | *smqnr13* | 16 | 2 | 1 |
| 14 | SMS009 | Sep-2016 | Emergency | + | + | + | + | + | + | 14.11 ± 0.07 | 9.59 ± 0.5 | 7.67 ± 0.2 | - | 16 | 2 | 1 |
| 15 | SMS011 | Sep-2016 | ICU | + | + | + | + | + | + | 26.76 ± 0.4 | 24.95 ± 0.6 | 6.22 ± 0.8 | *-* | 32 | 2 | 0.5 |
| 16 | SMS012 | Sep-2016 | Neurology | + | + | - | + | + | + | 1.37 ± 0.07 | 1.07 ± 0.3 | 0.65 ± 0.1 | - | 2 | 2 | 0.25 |
| 17 | SMS014 | Sep-2016 | Internal | + | + | + | + | + | + | 0.91 ± 0.2 | 0.94 ± 0.1 | 0.61 ± 0.1 | *smqnr30* | 2 | 2 | 0.25 |
| 18 | SMS017 | Sep-2016 | ICU | + | + | + | + | + | + | 0.99 ± 0.1 | 0.49 ± 0.03 | 0.40 ± 0.08 | *smqnr8* | 4 | 4 | 1 |
| 19 | SMS018 | Sep-2016 | Emergency | + | + | - | + | + | + | 3.28 ± 0.1 | 1.45 ± 0.2 | 0.89 ± 0.1 | New Variant 2 | 8 | 2 | 2 |
| 20 | SMI107 | Sep-2016 | Emergency | + | + | + | + | + | + | 1.41 ± 0.2 | 0.96 ± 0.1 | 0.83 ± 0.1 | - | 2 | 2 | 0.25 |
| 21 | SMI108 | Sep-2016 | ICU | + | + | + | + | + | + | 0.78 ± 0.1 | 0.25 ± 0.06 | 0.69 ± 0.2 | - | 1 | 1 | 0.5 |
| 22 | SMI110 | Sep-2016 | Emergency | + | + | + | + | + | + | 0.94 ± 0.1 | 0.68 ± 0.2 | 0.53 ± 0.09 | - | 2 | 2 | 0.25 |
| 23 | SMI112 | Sep-2016 | Emergency | + | - | - | + | + | + | 1.34 ± 0.08 | 0.75 ± 0.003 | 0.92 ± 0.1 | *smqnr8* | 2 | 2 | 0.5 |
| 24 | SMS052 | Oct-2016 | ICU | + | + | + | + | + | + | 0.43 ± 0.07 | 0.59 ± 0.05 | 0.44 ± 0.2 | - | 1 | 1 | 2 |
| 25 | SMS063 | Nov-2016 | Neurosurgery | + | + | + | + | + | + | 3.03 ± 0.8 | 2.73 ± 0.3 | 0.82 ± 0.1 | - | 2 | 0.25 | 0.25 |
| 26 | SMS080 | Nov-2016 | Glands | + | + | + | + | + | + | 13.59 ± 0.9 | 12.97 ± 0.8 | 4.33 ± 0.3 | - | 16 | 2 | 0.5 |
| 27 | SMV003 | Nov-2016 | Cardiothoracic surgery | + | + | - | + | + | + | 7.01 ± 0.4 | 6.66 ± 0.2 | 0.36 ± 0.09 | *smqnr11* | 4 | 1 | 0.25 |
| 28 | SMV004 | Nov-2016 | Internal hematology | + | + | + | + | + | + | 5.07 ± 0.6 | 7.65 ± 0.4 | 0.74 ± 0.1 | *smqnr11* | 4 | 1 | 0.25 |
| 29 | SMV005 | Nov-2016 | Emergency | + | + | + | + | + | + | 3.26 ± 0.8 | 2.67 ± 0.4 | 0.42 ± 0.1 | *smqnr8* | 2 | 0.5 | 2 |
| 30 | SMS091 | Dec-2016 | BMT^f^ | + | + | + | + | + | + | 5.88 ± 0.2 | 4.78 ± 0.6 | 0.78 ± 0.06 | *smqnr30* | 2 | 0.25 | 0.25 |
| 31 | SMS112 | Dec-2016 | ICU | + | - | + | + | + | + | 11.44 ± 0.3 | 10.71 ± 0.3 | 0.95 ± 0.2 | New Variant 3 | 8 | 2 | 0.25 |
| 32 | SMS122 | Dec-2016 | Blood | + | + | + | + | + | + | 63.73 ± 0.1 | 62.37 ± 0.8 | 2.28 ± 0.3 | - | 32 | 8 | 0.5 |
| 33 | SMS125 | Dec-2016 | Medical 2 | + | + | + | + | + | + | 13.51 ± 0.6 | 13.11 ± 0.1 | 4.30 ± 0.6 | - | 16 | 2 | 2 |
| 34 | SMS128 | Dec-2016 | Emergency | + | + | + | + | + | + | 1.50 ± 0.4 | 1.78 ± 0.1 | 0.71 ± 0.1 | *smqnr35* | 4 | 4 | 1 |
| 35 | SMS137 | Dec-2016 | Rheumatology | + | + | + | + | + | + | 13.32 ± 0.06 | 12.68 ± 0.2 | 0.68 ± 0.1 | - | 8 | 2 | 8 |
| 36 | SMV012 | Dec-2016 | Men's section | + | + | + | + | + | + | 11.93 ± 0.3 | 10.67 ± 0.4 | 0.75 ± 0.1 | - | 8 | 1 | 0.25 |
| 37 | SMV013 | Dec-2016 | Men's section | + | + | + | + | + | + | 0.95 ± 0.06 | 0.94 ± 0.1 | 0.89 ± 0.1 | *smqnr8* | 2 | 2 | 0.5 |
| 38 | SMI155 | Jan-2017 | Emergency | + | + | + | + | + | + | 1.34 ± 0.2 | 1.26 ± 0.06 | 0.78 ± 0.2 | - | 2 | 2 | 2 |
| 39 | SMS135 | Mar-2017 | Emergency | + | + | + | + | + | + | 101.41 ± 0.4 | 97.14 ± 0.7 | 9.25 ± 0.3 | New Variant 4 | 128 | 32 | 8 |
| 40 | SMV018 | Mar-2017 | Emergency | + | + | - | + | + | + | 10.13 ± 0.7 | 9.37 ± 0.4 | 0.99 ± 0.1 | *smqnr11* | 8 | 1 | 0.25 |
| 41 | SMV020 | Mar-2017 | Men's section | + | + | + | + | + | + | 11.67 ± 0.9 | 10.52 ± 0.4 | 0.67 ± 0.1 | *smqnr8* | 8 | 2 | 0.25 |
| 42 | SMV022 | Mar-2017 | ICU^e^ | + | + | + | + | + | + | 29.34 ± 0.9 | 23.43 ± 0.3 | 9.92 ± 0.3 | *smqnr9* | 32 | 4 | 8 |
| 43 | SMV029 | Mar-2017 | Emergency | + | + | + | + | + | + | 1.92 ± 0.3 | 1.88 ± 0.1 | 0.53 ± 0.09 | *smqnr11* | 2 | 2 | 2 |
| 44 | SMS142 | Apr-2017 | BMT | + | + | - | + | + | + | 13.75 ± 0.1 | 12.69 ± 0.2 | 0.89 ± 0.1 | - | 8 | 2 | 0.5 |
| 45 | SMS146 | May-2017 | Emergency | + | + | + | + | + | + | 0.82 ± 0.03 | 0.64 ± | 0.73 ± | *smqnr8* | 2 | 2 | 0.25 |
| 46 | SMV023 | May-2017 | Women's section | + | + | + | + | + | + | 6.55 ± 0.1 | 5.19 ± 0.4 | 0.27 ± 0.05 | - | 4 | 1 | 0.25 |
| 47 | SMV030 | Jun-2017 | Men's section | + | + | - | + | + | + | 0.90 ± 0.08 | 0.90 ± 0.02 | 0.45 ± 0.09 | *smqnr8* | 2 | 2 | 1 |
| 48 | SMI007 | NR | NR | + | + | + | + | + | + | 0.89 ± 0.06 | 0.57 ± 0.005 | 0.69 ± 0.1 | - | 2 | 2 | 0.25 |
| 49 | SMI019 | NR | NR | + | + | + | + | + | + | 5.61 ± 0.5 | 5.51 ± 0.2 | 0.95 ± 0.2 | - | 4 | 1 | 2 |
| 50 | SMI020 | NR | NR | + | + | + | + | + | + | 1.97 ± 0.1 | 2.44 ± 0.3 | 0.93 ± 0.2 | - | 4 | 4 | 0.25 |

^a^MIC; minimum inhibitory concentration, ^b^CIP; ciprofloxacin, ^c^Res; reserpine, ^d^LEV; levofloxacin, ^e^ICU; intensive care unit, ^f^BMT; bone marrow transplantation, ^g^NR; not recorded
