# Supplementary material for: Emergence of fluoroquinolone resistance and possible mechanisms in clinical isolates of Stenotrophomonas maltophilia from Iran
Source: Sci Rep. 2021 May 5;11:9582. doi: 10.1038/s41598-021-88977-z (PMC8100118; doi:10.1038/s41598-021-88977-z)
Supplement: Supplementary file 2 — Supplementary Figure S1. [file 41598_2021_88977_MOESM2_ESM.pdf]

Emergence of fluoroquinolone resistance in clinical isolates of *Stenotrophomonas maltophilia* from Iran: Possible mechanisms

Akram Azimi[1], Farhad Rezaei[2], Mehdi Yaseri[3], Sirus Jafari[4], Mohammad Rahbar[5], Masoumeh Douraghi*[1,6]

[1]Division of Microbiology, Department of Pathobiology, School of Public Health, Tehran University of Medical Sciences, Tehran, Iran.

[2]Virology Department, School of Public Health, Tehran University of Medical Sciences, Tehran, Iran.

[3]Department of Epidemiology and Biostatistics, School of Public Health, Tehran University of Medical Sciences, Tehran, Iran.

[4]Department of Infectious Diseases, Imam-Khomeini Hospital Complex, Tehran University of Medical Sciences, Tehran, Iran.

[5]Department of Microbiology, Reference Health Laboratories, Ministry of Health, Tehran, Iran.

[6] Food Microbiology Research Center, Tehran University of Medical Sciences, Tehran, Iran.

*** Corresponding author**

Masoumeh Douraghi, Ph.D., Division of Microbiology, Department of Pathobiology, School of Public Health, Tehran University of Medical Sciences, Poursina street, Enghelab-e-Eslami avenue, Tehran, Iran, PO Box: 14155-6446, Fax: +98 21 88954913 Tel: +98 21 42933152, Email: mdouraghi@tums.ac.ir

10 20 30 40 50 60 70 80 90 100

SmQnr 1 MESPMSPTVHRRLRIGADQYTGQKVVDQQFHECDFSGADLTGTEFINCSFYDADTRAGCRFNGATLKEASFRSCDISMCHFSFIKALGLEISECRAQGAD
SmQnr 2 ...T......K..............................A...........EN...
SmQnr 3 ...L......K....D.........................A............S.S........................N..................
SmQnr 4 ...L......K..............................A............S.S........................N..................
SmQnr 5 ...L.....HK..............................A............S.................G........N.........R........
SmQnr 6 ...L......K.....V.....................................S..........................N..................
SmQnr 7 ...L......K...S..........................N..................
SmQnr 8 ...L......K...S..........................N..................
SmQnr 9 ...L......K...S..........................N..................
SmQnr 10 ...L......K....S......................................S..........................N..................
SmQnr 11 ...L......K...S..........................N..................
SmQnr 12 ...L.....HK..............................A............S.................G....L...N..................
SmQnr 13 ...L..H...K..........................V................S.T........................N.V................
SmQnr 14 ...L......K..........R.T.................A............S.S........................N..................
SmQnr 15 ...T......K.........................SV...A...........ES.S........................N.V................
SmQnr 16 ...T......K.........................SV...A...........ES.S........................N.V................
SmQnr 17 ...L......K....S......................................S..........................N..................
SmQnr 18 ...L......K.....V..............D......................S..........................N..................
SmQnr 19 ...L......E..........D.........K......................S..........................N..N...............
SmQnr 20 ...L......E....................K......................S..........................N..N...............
SmQnr 21 ...L......K..............................A............S..........................N..................
SmQnr 22 ...L......K....S......................................S..........................N..................
SmQnr 23 ..........K...................Y..........A...........EN...
SmQnr 24 ...L.....................................A............S..........................N..................
SmQnr 25 ...L....A.K..............................A............S.S........................N..................
SmQnr 26 ...L......K...............Y...Q.......................S..............G...........N..................
SmQnr 27 ..........K.....V........................A...........EN...
SmQnr 28 ...T......K...................Y..........AS..........EN...
SmQnr 29 ..........K.....V........................A...........ENS...................L.....N..................
SmQnr 30 ...T......K...................Y..........A...........EN...
SmQnr 31 ...L......K..............................A............S.S........................N..................
smqnr 32 ...L......K.........P................V...A............S.S........................N..................
SmQnr 33 ...L..H...K..............................D............S.S........................N..................
SmQnr 34 ...L......K......H.......................A............S.S........................N..................
SmQnr 35 ...L......E....................K......................S..........................N..................
SmQnr 36 ...L......K....R......................................S...
SmQnr 37 ...L......E....................K......................S..........................N..................
SmQnr 38 ...L......T...S..........................N..................
SmQnr 39 ..........K...S.T...
SmQnr 40 ...L......K..........................V...A............S.S......T.................N..................
SmQnr 41 ...L.P....K....R......................................S..........................N..................

```
SmQnr 42             ...T.......K.........................V...A............S..........................N.V................
SmQnr 43             ...T..H....K.....N..................SV...A...........ES.S........................N.V................
SmQnr 44             ...T.......K........................SV...A...........ES.S........................N.V................
SmQnr 45             ...L.......E...................K......................S..........................N..................
SmQnr 46             ..FL...I...K........I.................................S..........................N..................
SmQnr 47             ...L.......K.........................V...A....S.......S.S......T.................N..................
SmQnr 48             ...L.......K...R....................................................................................
SmQnr 49             ...T.......K.....G............Y..........A...........EN.............................................
SmQnr 50             ...L.......E...................K......................S..........................N..................
SmQnr 51             ...L.......K.......H..................................S..........................N..................
SmQnr 52             ...L.......K..........................................S................H.........N..................
SmQnr 53             ...L.......E...................K......................S...........E..............N..................
SmQnr 54             ...L.......K..........................................S..........................N..................
SmQnr 55             ...L.......T..........................................S.............................................
SmQnr 56             .....F.....K..........................................S..........................N..................
SmQnr 57             ...L.......K..........................................S..........................N..................
SmQnr 58             .....F.....K..........................................S..........................N..................
SmQnr new variant 1  ...L.......E...................K......................S..........................N............H.....
SmQnr new variant 2  ...L.......K.........................V...A............S.S........................N..................
SmQnr new variant 3  ...L.......E..........E....H...K......................S..........................N..................
SmQnr new variant 4  ...L..H....K.........................V................S.T........................N.V................

                              110       120       130       140       150       160       170       180       190       200
                     ....|....|....|....|....|....|....|....|....|....|....|....|....|....|....|....|....|....|....|....|
SmQnr 1              FSNASFMNQITTRSWFCSAFIKKSNLRYANFSRVTLEKCELWENRWDGANVSGASFAGSDLSGGQFEGIDWNSANFTDCDLTRSELGELDLRSTNLRGAT
SmQnr 2              ...............................................................................N..N.................
SmQnr 3              ..................................................................................N.D...............
SmQnr 4              S.................................................................................N.D...............
SmQnr 5              ..................................................................................N.........T.......
SmQnr 6              ....................................................................V.............N.................
SmQnr 7              ........................K.........................................................N.................
SmQnr 8              ..................................................................................N.................
SmQnr 9              ..................................A...............................................N.................
SmQnr 10             ..................................A...............................................N.................
SmQnr 11             ....................................................................V.............N.................
SmQnr 12             ....N.............................................................................N.........T.......
SmQnr 13             ..G........P..G.....................................................V.............H.................
SmQnr 14             ..................................................................................N.D...............
SmQnr 15             ...........P..G........................................................S..........H.................
SmQnr 16             ...........P..G........................................................S.....G....H.................
SmQnr 17             ..................................................................................N.................
SmQnr 18             ....................................................................V.............N.................
SmQnr 19             ..................................................................................N.........R.......
SmQnr 20             ..................................................................................N.........R.......
SmQnr 21             ..S...........G...................................................................N.................
```

```
SmQnr 22              ..S...............................A...............................................N.................
SmQnr 23              ...............................................................................N..N.................
SmQnr 24              ...............................................................................N....................
SmQnr 25              ..................................................................................N.D...............
SmQnr 26              ..................................................................................N.................
SmQnr 27              .............................S.................................................N..N.................
SmQnr 28              ...............................................................................N..N.................
SmQnr 29              ..............G...................................................................N.................
SmQnr 30              ...............................................................................N..N.................
SmQnr 31              ..................................................................................N.D...............
smqnr 32              .................................A...........................................G.N..N.................
SmQnr 33              .........M.P..G...................................................................H.................
SmQnr 34              ..................................................................................N.D...............
SmQnr 35              ..................................................................................N.........R.......
SmQnr 36              ..............G...................................................................N.................
SmQnr 37              ..............G...................................................................N.........R.......
SmQnr 38              ..................................................................................N.................
SmQnr 39              ..................................................................................N.................
SmQnr 40              ..................................................................................N.D...............
SmQnr 41              ..............G...................................................................N.........R.......
SmQnr 42              ...........P..G..R.....................................................S.....G....H.................
SmQnr 43              ...........P..G..................A..................................V.............H.................
SmQnr 44              ...........P..G..............................................................G....H.................
SmQnr 45              ............................................................................S.....N.........R.......
SmQnr 46              ..................................................................................H.................
SmQnr 47              ..................................................................................N.D...............
SmQnr 48              ..............G...................................................................N..........A......
SmQnr 49              ...............................................................................N..N.................
SmQnr 50              ..................V...............................................................N.........R.......
SmQnr 51              ..................................A...............................................N.................
SmQnr 52              ....................................................................V.............N.................
SmQnr 53              ..............G..............................G....................................N.........R.......
SmQnr 54              ..................E...............................................................N.................
SmQnr 55              ..............G...................................................................N..........A......
SmQnr 56              ..................................................................................N.................
SmQnr 57              ....N.............................................................................N.................
SmQnr 58              ..................................................................................N.................
SmQnr new variant 1   ..................................................................................N.........R.......
SmQnr new variant 2   ..................................................................................N.D...............
SmQnr new variant 3   ..................................................................................N.........R.......
SmQnr new variant 4   ..G........P..G..............................................R.......V.............H.................

                               210
                      ....|....|....|....
SmQnr 1               LDVQQVALLMQRIGITVVP
```

```
SmQnr 2     ...................
SmQnr 3     ...................
SmQnr 4     ...................
SmQnr 5     ...................
SmQnr 6     ...................
SmQnr 7     ...................
SmQnr 8     ...................
SmQnr 9     ...................
SmQnr 10    ...................
SmQnr 11    ...................
SmQnr 12    ...................
SmQnr 13    ..L................
SmQnr 14    ..................A
SmQnr 15    ...................
SmQnr 16    ...................
SmQnr 17    ...................
SmQnr 18    ...................
SmQnr 19    ...................
SmQnr 20    ...................
SmQnr 21    ...................
SmQnr 22    ...................
SmQnr 23    ...................
SmQnr 24    ...................
SmQnr 25    ...................
SmQnr 26    ...................
SmQnr 27    ...................
SmQnr 28    ...................
SmQnr 29    ...................
SmQnr 30    ...................
SmQnr 31    ...................
smqnr 32    ...................
SmQnr 33    ...................
SmQnr 34    ...................
SmQnr 35    ...................
SmQnr 36    ...................
SmQnr 37    ...................
SmQnr 38    ...................
SmQnr 39    ...................
SmQnr 40    ...................
SmQnr 41    ...................
SmQnr 42    ...................
SmQnr 43    ...................
SmQnr 44    ...................
SmQnr 45    ...................
SmQnr 46    ...................
```

```
SmQnr 47             ...................
SmQnr 48             ...................
SmQnr 49             ...................
SmQnr 50             ...................
SmQnr 51             ...................
SmQnr 52             ..M................
SmQnr 53             ...................
SmQnr 54             ...................
SmQnr 55             ...................
SmQnr 56             ...................
SmQnr 57             ...................
SmQnr 58             ....X..............
SmQnr new variant 1  ...................
SmQnr new variant 2  ...................
SmQnr new variant 3  ...................
SmQnr new variant 4  ..L................
```

Supplementary Figure S1. Sequence alignment of the Sm*qnr* alleles. Numbers, dashes, and colored dots show the number of amino acids, the absence of amino acids, and the similarity of amino acids, respectively.
